# Selection occurs within linear fruit and during the early stages of reproduction in *Robinia pseudoacacia*

**DOI:** 10.1186/1471-2148-14-53

**Published:** 2014-03-21

**Authors:** Cun-Quan Yuan, Yu-Han Sun, Yun-Fei Li, Ke-Qi Zhao, Rui-Yang Hu, Yun Li

**Affiliations:** 1 National Engineering Laboratory for Tree Breeding; Key Laboratory of Genetics and Breeding in Forest Trees and Ornamental Plants, Ministry of Education, College of Biological Sciences and Technology, Beijing Forestry University, Beijing 100083, China; 2 Department of Ornamental Horticulture and Landscape Architecture, China Agricultural University, Beijing 100193, China; 3 Wenquan Nursery, Beijing Gardening and Greening Bureau, Beijing 100095, China

**Keywords:** Pollen donor composition, Inbreeding depression, Paternity analysis, *Robinia pseudoacacia*, Viability selection

## Abstract

**Background:**

Pollen donor compositions differ during the early stages of reproduction due to various selection mechanisms. In addition, ovules linearly ordered within a fruit have different probabilities of reaching maturity. Few attempts, however, have been made to directly examine the magnitude and timing of selection, as well as the mechanisms during early life stages and within fruit. *Robinia pseudoacacia*, which contains linear fruit and non-random ovule maturation and abortion patterns, has been used to study the viability of selection within fruit and during the early stages of reproduction. To examine changes in the pollen donor composition during the early stages of reproduction and of progeny originating from different positions within fruit, paternity analyses were performed for three early life stages (aborted seeds, mature seeds and seedlings) in the insect-pollinated tree *R. pseudoacacia*.

**Results:**

Selection resulted in an overall decrease in the level of surviving selfed progeny at each life stage. The greatest change was observed between the aborted seed stage and mature seed stage, indicative of inbreeding depression (the reduced fitness of a given population that occurs when related individual breeding was responsible for early selection). A selective advantage was detected among paternal trees. Within fruits, the distal ends showed higher outcrossing rates than the basal ends, indicative of selection based on the order of seeds within the fruit.

**Conclusions:**

Our results suggest that selection exists both within linear fruit and during the early stages of reproduction, and that this selection can affect male reproductive success during the early life stages. This indicates that tree species with mixed-mating systems may have evolved pollen selection mechanisms to increase the fitness of progeny and adjust the population genetic composition. The early selection that we detected suggests that inbreeding depression caused the high abortion rate and low seed set in *R. pseudoacacia*.

## Background

The potential for competition and selection during zygote formation and seed maturation has been characterised in flowering plants [1,2], and in many angiosperms, pollen selection mechanisms that reduce inbreeding depression and promote outcrossing have evolved [3]. Pollen competition acts as a post-pollination mechanism responsible for the highly heterogeneous patterns of male reproductive success among embryos and in later life stages [4]. Viability (early) selection, which is responsible for the significant number and high mortality of fertilised ovules during the earliest stages of the life cycle, plays an important role in shaping the genetic composition of plant populations [4,5].

Among these early life stages, the period of seed ontogeny between fertilisation and seed maturity is perhaps the most critical phase of a plant’s life cycle for determining reproductive yield and progeny vigour [3,6].

Selection component analysis has been applied in several recent studies to measure viability selection in populations. This technique revealed a strong viability component of selection in annual species [7-9] and perennial trees [3-5]. For example, Hasegawa et al. [3] characterised the process of pollen selection during the early stages of reproduction using microsatellite genotyping of pollen grains and mature seeds, and found that the self-rate dropped from 90.2% during the pollination stage to 0.3% during the seed stage, suggesting that the mechanism of self-incompatibility strongly avoids self-pollen before seed production in *Castanea crenata*. Hufford and Hamrick [5] determined the genetic composition of three early stages in *Platypodium elegans* and found that the greatest change in the genetic composition of progeny occurred between mature seeds and established seedlings, suggesting that inbreeding depression was responsible for early selection. Selection component analysis is typically applied to only one life stage (e.g. the majority of selection component studies have focused on the transition between seedlings and adults) collected during a single reproductive year or, rarely, the same life stage collected in multiple years. However, viability selection may occur at any point from fertilisation to reproductive maturity. To fully characterise viability selection, paternity analysis should be performed for successive life stages. Viability selection may play an important role in male reproductive success; however, scant attention has been paid to this issue.

Typically, self-incompatibility and inbreeding depression are believed to result in selective differences in survival during the earliest life-cycle stages [10,11]. For self-incompatibility, only outcross-pollen grains are used for seed production [3,11,12]. For late-acting self-incompatibility, selection against self-fertilised ovules occurs prior to seed maturation [5]. Inbreeding depression; i.e. the reduction in fitness under inbreeding, arises due to an accumulation and expression of deleterious mutations (i.e. genetic load), and possibly overdominance effects, at loci linked to fitness [13,14], and fitness differences due to inbreeding depression may affect progeny survival at any stage in the life cycle [5]. Models and empirical observations indicate that high levels of inbreeding depression are experienced early in the life cycle in predominantly outcrossing species [15,16]*.* Furthermore, inbreeding avoidance, in which pollen grains from more distant and non-related parents are more likely to produce seeds [17-19], and mate-quality factors, such as pollen tube competition, and the different growth rates of fertilised ovules [20], also play important roles in shaping the genetic composition of a population. As a result, these systems may cause differences in pollen donor compositions among the aborted seed stage, seed stage and seedling stage.

However, selection may not occur during the early life stages, but instead within an inflorescence or ovule within linear fruits. Several studies have shown that fertilisation, resource gradients and/or spatial advantages exist within a plant during seed maturation depending on the time of initiation and relative position of a flower within an inflorescence or ovule in linear fruits [6,21-24]. For example, a study on ovule developmental patterns in *Bauhinia ungulata* showed that non-fertilised and early aborted ovules were often located near the basal end of the ovary. Mature seeds were found mainly in the stylar half of fruits, where ovules are likely to be fertilised by fast-growing pollen tubes.

Several hypotheses have been proposed to explain the non-random maturation pattern and high levels of seed abortion observed in most plant species. Many seeds may be aborted due to the expression of lethal or deleterious alleles. Additionally, high levels of homozygosity due to inbreeding may lead to high seed abortion rates [25]. Also, abortion may depend on the supply of resources for seed development [21], or seed abortion may be due to differences in the ability of the developing seeds to gather maternal resources [26]. Although the effect of ovule position on seed maturation patterns and abortion in plants has been well studied, its inherent influence at the molecular level remains unclear. To explore possible effects of ovule position on offspring fitness, investigating the effects of ovule position on male reproductive success is necessary.

*Robinia pseudoacacia*, commonly known as the black locust, is a tree in the subfamily Faboideae of the pea family Fabaceae. It has linear, unicarpellate and multiovulate fruits containing 9–26 ovules generally arranged alternately along one suture [6]. Normally, fruit and seed sets are low in *R. pseudoacacia*, and the majority of ovules abort and fail to develop within multi-seeded fruits. However, the distribution of ovules that reach maturity as viable, fully developed seeds is non-random in fruits [6]. These features allow us to investigate selection during early life stages and within fruit.

In this study, we conducted a paternity analysis in a population of *R. pseudoacacia* using microsatellite markers to evaluate the process of selection during the early stages (aborted seed stage, mature seed stage and seedling stage) of reproduction. We also investigated the pollen donor composition of seeds (ovules) at different positions within the fruit to evaluate the effect of ovule position on the genetic composition of progenies.

Here, we specifically addressed the following questions:

1. Does the genetic composition differ among aborted seeds, mature seeds and seedlings in *R. pseudoacacia*?

2. If so, which factors are responsible for this viability selection?

3. Does the genetic composition of seeds (ovules) that are linearly ordered within a fruit differ between different positions of a fruit?

4. If so, which positions show the highest outcrossing rate, and what might account for any differences?

5. What factors caused seed abortion in *R. pseudoacacia*?

Ultimately, we estimated the effects of selection on shaping the genetic composition of *R. pseudoacacia*.

## Results

### Paternity and gene flow

The genetic variation of the six microsatellite loci among the samples was large (see Additional file 1). The mean number of alleles per locus and mean expected heterozygosity were 15.67 and 0.7669, respectively. The total exclusion probability for the second parent, corresponding to the total exclusionary power for the pollen parent, was 0.9957, which allowed precise paternity analysis. Paternity assignment using simple exclusion resulted in one to five possible parents for each progeny assigned to pollen donors within the study area. Of 720 offspring analysed, 538 were assigned to a single pollen parent. For 105 offspring, no adults within the study site had positive LOD scores, indicating that the fathers of these offspring were outside the research site.

Among the 720 offspring, 632 were products of outcrossing, and the pollen donors of 527 were within the research site. A total of 152 (59.14%) of 257 adults produced offspring. The frequency distribution of the total number of outcrossed offspring produced by each paternal parent pooled for all three life stages indicated significant differences in the success of individual pollen donors. Each paternal parent produced a mean of 3.47 offspring. However, some pollen parents produced as many as 10–38 offspring.

**Figure 1:**
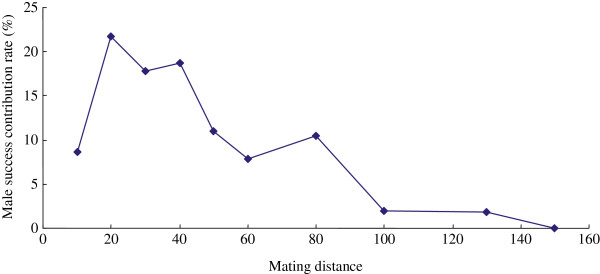
Relationships between the male success contribution rate and their mating distance from known female trees.

Relationships between reproductive success of male parents and their mating distance to known female trees were estimated. Analyses were conducted using the progeny that had a pollen donor within the study plot. The effective pollen movement ranged from 0 to 100 m; the largest pollen movement observed was 128 m within this research site. The effective mating distance was within 0–80 m; within this area, the pollen contribution rate approached 96.26%. Overall, the mating probability decreased and the pollen flow weakened as the mating distance increased. Correlation analysis showed that the reproductive success of male parents was negatively associated with mating distance (*r* = −0.757, *p* = 0.018; Figure 1), supporting the idea that as the mating distance increased, the mating probability decreased.

### Differences in pollen donor composition and male reproductive success at three early life stages

Of the 720 progeny analysed, 14.58% (105) could not be assigned to a local pollen donor and were considered to be the result of gene flow. The results of the ANOVA comparing the total number of progeny resulting from outside pollen donors (immigrant pollen) indicated significant differences among the three early life stages (*p* = 0.042) and among trees (*p* = 0.019). The number of progeny produced by immigrant pollen decreased from the aborted stage to the seedling stage (aborted stage, 10.40 ± 6.73; mature seed stage, 5.80 ± 3.70; seedling stage, 4.80 ± 3.42).

When comparing the number of effective pollen donors within research sites that produced at least one progeny among the three early life stages, the seedling stage (29.2 ± 2.7) had a significantly higher total number of effective pollen donors than the aborted seed stage (19.0 ± 2.0) and mature seed stage (21.6 ± 4.2; *p* = 0.001). The difference was also significant among trees (*p* = 0.010). In addition, using ANOVA to compare the number of progeny produced by effective paternal parents revealed significant differences among life stages (*p* = 0.015) when selfed progeny were included in the data set; the seedling stage showed the lowest value (abortion stage, 1.8805 ± 0.2837; mature seed stage, 1.9055 ± 0.3025; seedling stage, 1.4352 ± 0.1196). When selfed progeny were removed from the data, significant differences were also observed (*p* = 0.025); however, the abortion stage showed the lowest value (abortion stage, 1.3084 ± 0.4208; mature seed stage, 1.7846 ± 0.2010; seedling stage, 1.4161 ± 0.1652).

The distance of pollen movement (average mating distance) inferred based on the distance between the maternal trees and the pollen donors within the research site, showed a significant difference among maternal trees irrespective of whether the selfed progeny were pooled (*p* < 0.0001), or not pooled (*p* < 0.0001). The ANOVA to compare the mean distance of pollen movement was not significantly different among life stages (Aborted seeds, Mature seeds and Seedling) when self-fertilized progeny were included in the data set and assigned a pollen dispersal distance of 0 (*p* = 0.401). However, the average mating distance was significantly different among early life stages when the selfed progeny were removed from the data (*p* = 0.014). The abortion stage showed the highest average mating distance (abortion stage = 38.879 ± 23.383 m; mature seed stage = 31.902 ± 23.034 m; seedling stage = 33.167 ± 21.223 m). However, because the progeny resulting from pollen that originated outside of the study area were not included, the actual mean distances of pollen movement will be much longer than the within population means given here.

The above results suggest that selective advantages exist among paternal trees.

### Differences in pollen donor composition and male reproductive success at four positions within fruit

ANOVA comparing the total number of progeny from outside pollen donors (immigrant pollen) indicated no significant differences among the four positions within fruit (*p* = 0.452) or among trees (*p* = 0.090). ANOVA to compare the number of progeny per effective paternal produced showed no significant differences between different positions whether selfed progeny were included (*p* = 0.457) or removed (*p* = 0.446) from the data set.

ANOVA results showed that the average mating distance differed significantly among maternal trees whether the selfed progeny were pooled (*p* < 0.0001) or not pooled (*p* < 0.0001). However, the average mating distance did not differ significantly among different positions within fruit whether selfed progeny were pooled (*p* = 0.956) or removed (*p* = 0.981).

### Genetic diversity and outcrossing

Pooled diversity measurements are reported in Table 1. The six microsatellite loci were highly polymorphic, including high heterozygosity values (57–75%) and allele counts (mean of 11.625). Observed heterozygosity was less than expected for adults and at each life stage pooled across the five trees, indicative of a slight heterozygote deficit at all stages in this population. Inbreeding coefficients decreased across three successive life stages. Levels of inbreeding were highest in the aborted seed populations and lowest in the seedling population (Table 1).

**Table 1:** Genetic diversity of the adult (parent) and offspring populations

**Life stage**	**Sample size**	***N***_**A**_	***N***_**E**_	***H***_**O**_	***H***_**E**_	***F***_**IS**_
Adults	257	12.3333 ± 3.4448	5.5003 ± 2.2874	0.6240 ± 0.1833	0.7868 ± 0.1000	___
Aborted seeds	240	12.6667 ± 3.0111	4.5553 ± 1.4236	0.5772 ± 0.1732	0.7632 ± 0.0769	0.2446
Mature seeds	240	9.8333 ± 1.7224	3.0333 ± 0.7872	0.6118 ± 0.2215	0.7345 ± 0.0759	0.1594
Seedlings	240	11.6667 ± 3.9328	4.5276 ± 1.3957	0.7525 ± 0.1927	0.7617 ± 0.0749	0.0487

Note: *N*_A_: actual number of alleles; *N*_E_: effective number of alleles; *H*_O_: observed heterozygosities; *H*_E_: expected heterozygosities; *F*_IS_: coefficient of breeding.

Estimates of selfing were calculated for each maternal tree and pooled for each life stage (Table 2). The results showed that selfing rates were highest during the aborted seed stage and lowest during the seedling stage, revealing a trend of increasing outcrossing rates across life stages (see Additional file 2). ANOVA results showed that the differences among the three early stages were significant (*p* = 0.012). The selfing rate at the aborted seed stage (25.42 ± 13.85%) was significantly higher than that at the mature seed stage (8.75 ± 9.13%, *n* = 5, GLM, *p* = 0.020). No apparent difference was observed between the mature seed stage (8.75 ± 9.13%) and seedling stage (4.17 ± 3.90%, *n* = 5, GLM, *p* = 0.476), despite the decreasing selfing rate. However, the results showed significant differences between the aborted seed stage (25.42 ± 13.85%) and seedling stage (4.17 ± 3.90%, *n* = 5, GLM, *p* = 0.005).

**Table 2:** Number of self offspring at three stages from different maternal trees

	**Aborted seeds**	**Mature seeds**	**Seedling**
**Maternal tree**	**Self (no.)**	**Self (no.)**	**Self (no.)**
MT 2	0.0833 (4)	0.000 (0)	0.000 (0)
MT 4	0.2500 (12)	0.0833 (4)	0.0625 (3)
MT 7	0.3333 (16)	0.1458 (7)	0.0625 (3)
MT 12	0.4375 (21)	0.2083 (10)	0.0833 (4)
MT 17	0.1667 (8)	0.0000 (0)	0.0000 (0)
Total	(61)	(21)	(10)
Average rate	0.2542 ± 0.1385	0.0875 ± 0.0913	0.0417 ± 0.0390

Estimates of outcrossing were calculated for each position within fruit (Table 3; see Additional file 3). The distal ends (0.6169) showed a higher outcrossing rate than the basal ends (0.3831). ANOVA results revealed differences between the distal and basal ends that were highly significant (*p* < 0.0001). Furthermore, the results also showed that differences among the four positions were highly significant (*p* = 0.002). Results of multiple comparison among the four positions revealed that the outcrossing rate of position A was significantly higher than those of the other positions.

**Table 3:** Outcrossing rate at four positions within fruit

**Position**		**Stage**			**Average**	
		**Aborted seeds**	**Mature seeds**	**Seedlings**		
Distal ends	A	0.8246	0.9048	0.9000	0.8765 ± 0.0450a	0.6169
	B	0.8070	0.7143	0.7000	0.7404 ± 0.0581b	
Basal ends	C	0.6491	0.6667	0.7000	0.6719 ± 0.0337b	0.3831
	D	0.7193	0.7143	0.7000	0.7112 ± 0.0100b	

Average values followed by the same letter were not significantly different according to Tukey’s multiple comparison test (P ≤ 0.05).

## Discussion

### Viability selection at three early life stages

In this study, the outcrossing rate during the seedling stage was estimated to be 95.83%, indicating that *R. pseudoacacia* was a predominantly outcrossing species. This result is consistent with Surles et al. [27], who estimated the outcrossing rate using allozyme analysis across 23 seed resources. However, the outcrossing rate at the seedling stage (95.83%) in our study was mach higher than estimated for most animal-pollinated tree species and by Surles et al. [27] (83%). Previous studies have shown that outcrossing rates of entomophilous tree species are typically less than 0.90 and sensitive to fluctuations in pollinator behaviour and stand structure [27-29]. For example, outcrossing rates for *Eucalyptus citriodora*, *E. delegatensis*, *E. stoatei*, *E. pauciflora* and *Platypodium elegans* were 0.85, 0.77, 0.82, 0.84 and 0.87, respectively [5,30-33]. However, the outcrossing rate observed in this study during the seedling stage was similar to the value estimated in *Alnus crispa* (0.95) [34]. We hypothesised that the high density of reproductive adults and pollinator behaviour may be important factors. In the present study, the space between each adult was small and the density was high. We observed that each maternal tree was surrounded by synchronous flowering trees during the flowering period at the present study site. The overlap of flowering periods between maternal and paternal flowers may encourage pollen transmission by bee pollinators from nearby trees, ultimately encouraging outcrossing. Previous studies on tropical species have indicated that a low density of flowering individuals encourages selfing. For tropical species pollinated by insects, the densities of reproductive adults play an important role in the pattern of mating systems [35-37]. These survey results supported our explanation of the high outcrossing rate in our population. In addition, the different sampling strategy might have contributed to the difference in the outcrossing rate, and our inclusion of viable seeds from all parts of the pods might have enhanced the outcrossing rate. Furthermore, the different times at which samples were taken (4 weeks after germination in Surles (1990); 10 weeks after germination in our study) and the different methods used for estimation of the outcrossing rate in the two studies (Allozyme analysis in Surles (1990); microsatellite analysis in our study) might also have contributed to the difference in the outcrossing rate.

However, these values represent only post-selection estimates rather than the outcrossing rate at fertilisation. Furthermore, we observed that some self-pollen grains adhered to the pistil before flowering in *R. pseudoacacia*, and that most pollinators moved frequently between individual trees. These characteristics may increase apparent selfing. Based on these facts, selection may have occurred during the early life stages in *R. pseudoacacia*.

In the present study, the outcrossing rate during the aborted seed stage was 74.58%, while this value increased to 91.25% and 95.83% during the mature seed stage and seedling stage, respectively. ANOVA results showed that differences among the three early life stages were significant. These results indicate that the selfing rate decreased and outcrossing increased over time. This is consistent with the trends observed for inbreeding coefficients, as well as heterozygosity for pooled data of the three life stages. In each case, inbreeding decreased and heterozygosity increased over time, suggesting that homozygosity should decrease and heterozygosity should increase across the three early life stages, which indicates viability selection at three early life stages. Significance testing results suggest that the most intense selection period was during the transition between the aborted seed stage and mature seed stage (*p* = 0.020). These data suggest that the effect of paternity on survival at three life stages is partially confined to self-fertilised progeny, which is possibly a consequence of inbreeding depression. Distinguishing between late-acting self-incompatibility and early acting inbreeding depression is difficult [11]. However, previous studies have argued that late-acting self-incompatibility can be explained by the action of early acting inbreeding depression effects due to lethal recessives [38].

Controlled pollination experiments and germination tests showed that progenies from cross-pollination treatments showed significantly higher fitness (seed set, seed mass, seedling emergence) than progenies from self-pollination, which resulted in high levels of inbreeding depression in *R. pseudoacacia*. Inbreeding depression between the fertilisation and seed maturation stages was estimated to be 0.5419, while that for the seedling emergence stage was 0.3654. The cumulative inbreeding depression (δ) across the mature seed stage, seedling emergence stage and seedling growth at 20 weeks was an average of 0.7452, indicating that inbreeding depression plays an important role in the maintenance of reproductive traits that promote outcrossing in *R. pseudoacacia*[39]. These results further support our finding that inbreeding depression was responsible for selection during the early life stages of *R. pseudoacacia*.

Although a dramatic decrease in the level of surviving selfed progeny from seeds to seedlings was seen, significance testing showed no apparent difference. Since collecting sufficient established seedlings in the field is difficult, these studies must be performed under experimental conditions. We believe that nursery experiments in the glasshouse rather than under natural conditions contribute to these discrepancies. The deleterious impacts of inbreeding depression are substantially greater in the field than under experimental conditions [40].

### Pollen donor composition differences at different positions within fruit

The effect of ovule position on seed maturation patterns and abortion in plants has been studied for several decades, and non-random seed (ovule) abortion and maturation within fruit has been characterised [6,22-25]. Our previous study on ovule developmental patterns in *R. pseudoacacia* from two populations showed that nearly two-thirds of ovules within fruit could not develop to mature seeds, but instead were non-fertilised or aborted. The majority of ovules at nearly all positions within a fruit were aborted. However, non-fertilised and aborted ovules were found more often near the basal end of the ovary, while mature seeds were found mainly in the stylar half of fruits [39]. Such patterns are consistent with the maturation patterns detected in leguminous species [41]. Several hypotheses have been proposed to explain the non-random maturation pattern and high levels of seed abortion in most plant species. Some argue that many seeds may be aborted due to the expression of lethal or deleterious alleles, and a high level of homozygosity due to inbreeding may lead to high seed abortion rates. Under these conditions, abortion may be random within fruit [25]. It is also possible that abortion depends on the supply of resources for seed development; according to this hypothesis, the ovules (zygotes) at the basal ends are more likely to develop into mature seeds. In contrast, ovules on the distal ends are likely to abort since they are farther from the supply of resources [21]. Furthermore, yet others suggest that seed abortion may be due to differences in the ability of the developing seeds to gather maternal resources. Based on this hypothesis, stronger seeds may obtain resources more efficiently than less-competent seeds (which will ultimately starve). According to this hypothesis, seed abortion is the consequence of gametophytic competition for access to ovules [26]. A basipetally decreasing sequence of ovule fertilisation has been reported for several species (*Phaseolus coccineus*, Rocha and Stephenson [42]; *Sophora japonica*, O’Donnell and Bawa [43]; *B. ungulata*, Menaali and Rocha [22]; *R. pseudoacacia*, Susko [6]; *Hesperis matronalis*, Susko and Clubb [24]). Typically, early fertilisation of distal ovules and failure of basal ovules to develop into seeds are attributable to fast-growing pollen tubes and the early initiation of distal ovules, which gives them a temporal “head start” and allows them to develop into stronger regional sinks for resources.

In the present study, a significantly higher outcrossing was detected in the distal ends than the basal ends of the fruit. Of all the progenies analysed using paternity analysis, the rate of selfed progeny originating from the basal ends (0.6169) was higher than that at the distal ends (0.3831), indicative of sexual selection within fruit. In the environment, we observed that outcross-pollen tubes grow faster than self-pollen tubes in *R. pseudoacacia*. Thus, if pollination was performed at the same time, distal ovules would be fertilised earlier following cross-pollinations than self-pollinations due to its proximity to the pollen entrance. If insect-visiting activity is not frequent (outcross-pollen is limited), distal ovules would be fertilised preferentially by outcross-pollen. Compared with the distal ovules, the basal ovules are expected to be fertilised by reduced amounts of growth pollen, or even autologous pollen. In this case, homozygosity levels may be higher and seed abortion may increase from the basal to distal ends as a result of inbreeding depression. On the basis of the above research results, we concluded that non-random abortion and maturation of ovules in *R. pseudoacacia* was due to the early initiation of distal ovules due to fast-growing pollen tubes and inbreeding depression. We assumed that distal ovules are likely to be fertilized earlier and prior to be fertilized by fast-growing pollen tubes than basal ovules, and the early initiation of distal ovules may give them a “head start” and allow them to develop into stronger regional sinks for maternal resources than less competent ovules (which will ultimately starve). This fertilisation gradient could increase the probability of fertilisation by self-pollination in basal ovules compared to distal ends, which could increase homozygosity. Thus, seed abortion may increase from distal to basal ends as a result of inbreeding depression. Overall, the species showed non-random abortion and maturation within fruit.

### Outcrossing in *R. pseudoacacia*

In most plants, pollen transmission by biological and abiological vectors plays an important role in siring. However, not all pollen that adheres to the pistil can produce seeds [44]. In natural ecosystems, plants have evolved many mechanisms to avoid inbreeding and encourage outcrossing, such as self-incompatibility [11,12] and inbreeding avoidance [17-19].

In *R. pseudoacacia*, the physical separation of stigmatal and antheral surfaces, as well as the protogynous flowering habits, encourage outcrossing [27]. This mechanism cannot effectively prevent geitonogamy, but selfing may be high in plants with mass flowers and asynchronous flowering because pollinators may forage longer among flowers of the same plant [45,46]. In this case, self-pollination may occur in *R. pseudoacacia.* Furthermore, hand-pollination experiments demonstrated that *R. pseudoacacia* can obtain fruits through autogamy and geitonogamy [39]. Thus, *R. pseudoacacia* may have evolved mechanisms to maintain high fitness levels.

A comparison of pollen tube growth between self-pollen and outcross-pollen indicates that outcross-pollen grows faster than self-pollen, which takes precedence over self-pollen in fertilisation of the ovules when both pollen grains are present simultaneously [39]. When comparisons were made between local and immigrant pollen at different life stages, significant differences were observed between three stages (Aborted seeds, Mature seeds and Seedling) for both the total number of progeny from outside pollen donors (immigrant pollen) and the effective pollen donors within the research site that produced at least one progeny. In addition, differences were observed in the number of progeny per effective paternal produced. These results suggest that a selective advantage existed among paternal trees, which may increase the quality of offspring. In addition, selective abortion and maturation of ovules based on the order of fertilisation within fruit may enhance the mean quality of resulting progenies. Furthermore, selection resulted in an overall decrease in self-fertilised progeny across each life stage, which suggests that inbreeding depression promoted outcrossing in *R. pseudoacacia* by acting as a post-pollination barrier to selfing during early life stages. The above factors may result in an apparently outcrossed progeny population, which maintains a high genetic diversity.

Overall, early selection in *R. pseudoacacia* suggests that gene-flow events are important for this species. Moreover, it also suggests that viability selection may play a significant role in adjusting the population genetic composition of self-compatible trees and trees with mixed-mating systems. The early selection that we detected suggests that inbreeding depression is responsible for the high abortion rate and low seed set in *R. pseudoacacia*.

## Conclusions

We investigated the pollen donor composition of three early life stages and different ovule positions within fruit in an insect-pollinated tree, *R. pseudoacacia*. Using microsatellite genotyping analysis, we showed an overall decrease in the level of surviving selfed progeny across each life stage, with the greatest change occurring between the aborted seed stage and mature seed stage, indicating that inbreeding depression was responsible for early selection. During crossing events, a selective advantage was detected among paternal trees during early life stages. Selfed progeny showed a significant decline from the basal to distal ends within fruit, with the distal ends showing a higher outcrossing rate than the basal ends, indicative of selection based on the order of seeds within fruit. Our research suggests that selection occurs both in linear fruit and during the early stages of reproduction, and that these selections can affect male reproductive success during the early life stages. This indicates that tree species with mixed-mating systems may have evolved pollen-selection mechanisms to increase the fitness of progeny and adjust the population genetic composition. The early selection that we detected indicates that inbreeding depression is responsible for the high abortion rate and low seed set in *R. pseudoacacia*. To our knowledge, this is the first study to explore selection within linear fruit during the early life stages of a plant species based on microsatellite genotyping.

## Methods

### Study site and sample collection

We established a 5-ha plot (200 × 250 m) at the Mijiabu tree farm (40°30′302″ N, 116°00′015″ E) in Yanqing, Beijing, China, in which all adult trees were mapped. We considered all trees as potential pollen donors in this study (*n* = 257).

**Figure 2:**
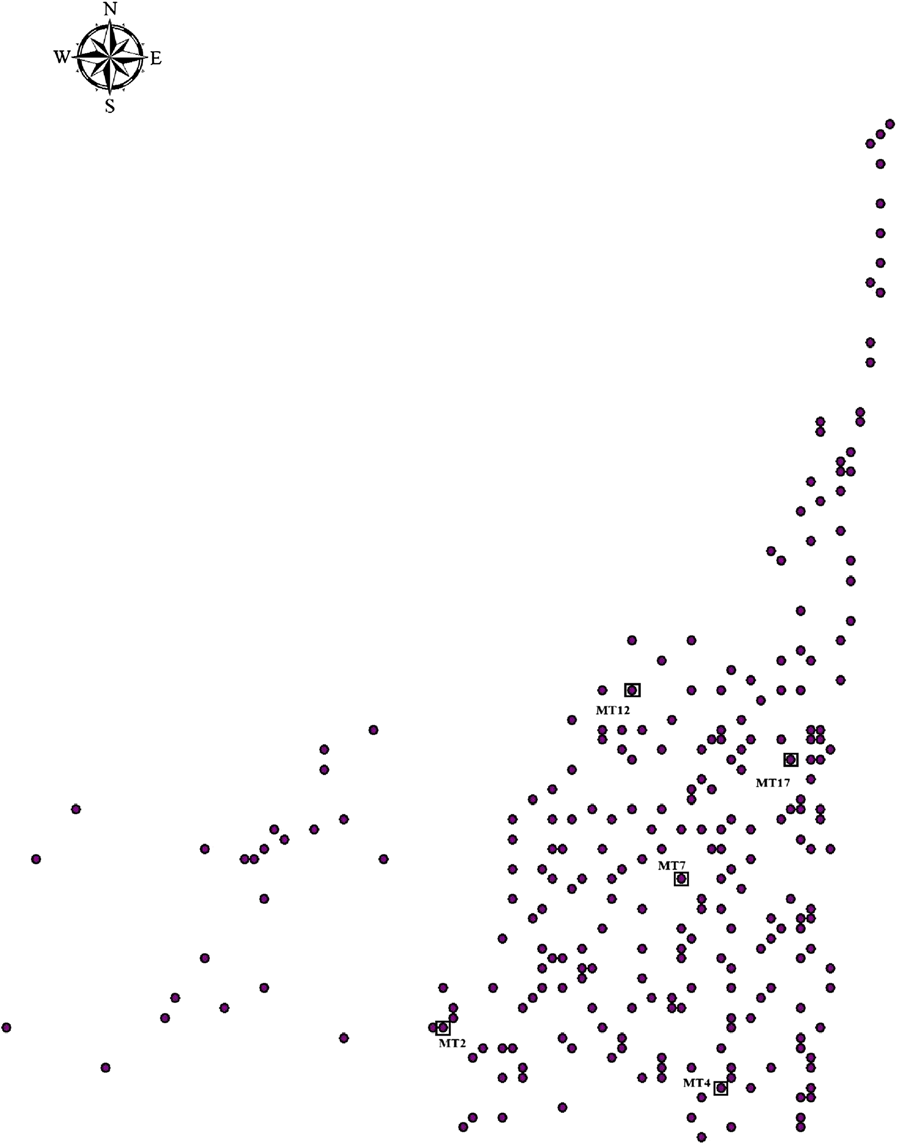
**Location of the five *Robinia pseudoacacia *maternal trees and the potential pollen trees.** Maternal trees are represented by black boxes with ID numbers. The potential pollen trees are represented by filled circles.

Leaf tissues were collected from each of the 257 trees. Five adult trees, with sufficient fruits for sampling, were selected as maternal trees for collection of the progeny (Figure 2). In total, 100 mature fruits from each maternal tree were collected in October 2011. Each fruit was divided into four positions (A, B, C and D), proceeding from the distal end to the basal end of the fruit. All seeds (ovules) at each position were collected from each fruit. Each seed (ovule) was classified according to its condition as a mature or aborted seed. Aborted seeds were easily identified because they showed signs of growth and were always necrotic; these ovules were much smaller than a fully mature seed. Mature seeds were brown–black in colour and plump, with a completely formed seed coat (the seed pattern was formed according to Susko [6]). Finally, 12 aborted seeds were randomly selected from each position per maternal tree and treated as aborted stage seeds. Twelve mature seeds were randomly selected from each position per maternal tree and treated as mature-stage seeds. Another 100 mature seeds were selected randomly from each position per maternal tree; these seeds were germinated in the glasshouse. After 10 weeks, 12 seedlings that originated from seeds from each position of all maternal trees were selected randomly, and leaf tissues were collected from each of the seedlings and treated as seedling-stage samples. In total, each stage was represented by 240 samples (*n* = 240, 4 positions × 5 maternal trees × 12 samples).

### DNA extraction and genotyping

Mature and aborted seeds were ground in Eppendorf tubes and soaked with pre-treatment solution for 24 h. Total DNA from seed tissues was then isolated using the Plant DNA Extraction Kit (Tiangen Biotech, Beijing, China) according to the manufacturer’s protocol.

Six SSR primers were selected and labelled with a fluorescent dye for the paternity analysis (Table 4) [47-49]. Polymerase chain reaction (PCR) amplification was performed using an ABI 9700 thermal cycler in a 12.5-μL reaction mixture containing 30 ng of genomic DNA, 1× Taq buffer, 2.5 mM MgCl_2_, 0.25 mM dNTPs, 0.20 μM of each primer pair and 0.5 units of Taq DNA polymerase. The amplification reaction was performed with the following two procedures: first, an initial denaturation step at 94°C for 5 min, followed by 10 cycles at 94°C for 30 s, T_m_ for 30 s (decreasing at n°C per cycle, n = (T_mmax_ – T_mmin_)/10), 72°C for 90 s, 20 cycles at the annealing temperature and a final extension at 72°C for 10 min and second, an initial denaturation step at 94°C for 5 min, 35 cycles at 94°C for 30 s, T_m_ for 30 s, 72°C for 90 s and a final extension at 72°C for 10 min.

**Table 4:** **Sequences and fluorescent labels of primers for six microsatellites of *Robinia pseudoacacia***

**Locus**	**Fluorescence label**	**Motif**	**Primer sequence**	**Size range (bp)**	**T**_**m **_**(°C)**	**GenBank accession no.**
Rops08	HEX	(CA)_8_TA(CA)_3_	TTCTGAGGAAGGGTTCCGTGG	192–212	63 to 52	AB075033
			GTTAAAGCAACAGGCACATGG			
Rp206	HEX	(GT)_9_	GCCAAATCCCATTAGATCACAGTTGA	200–232	65 to 58	AB353932
			AGAAGTTAGACTTACGTGCTGC			
Rops05	FAM	(AC)_2_GC(AC)_7_	TGGTGATTAAGTCGCAAG	114–148	56	AB075031
			GTGGTTGTGACTTGTACGTAAGTC			
Rops06	FAM	(GT)_3_ACA(GT)_11_	CTAAGGAGGTGCTGACCCTC	114–146	65 to 58	AB075032
			TTAATCTGTGATGGGACACTG			
Rp109	TAMRA	(AG)_17_	GAGGAATCACAAAACCGTTTGG	119–151	65 to 63	AB353930
			TGGGATTTGAGAGAGTGGTGGTG			
Rp200	TAMRA	(AG)_23_	GGTTTCTTTGTTCACCTGCTCTGG	160–185	65 to 60	AB353933
			ACCTACGTGTCCACGGCTCT			

PCR products were electrophoresed on an ABI 3100 Genetic Analyser (Applied Biosystems, Foster City, CA, USA), and allele sizes were determined using the fragment analysis software packages GeneScan 3.0 and Genotyper 2.1 (Applied Biosystems).

### Paternity analysis

The paternity of each offspring was assigned by comparing the genotypes of adults and offspring using the CERVUS 3.0 software [50], based on a maximum-likelihood paternity assignment. The simulation parameters for CERVUS were as follows: 10,000 tests were performed with a total of 257 individuals present in the study plot; 88% of the candidate parents were sampled and 100% of the loci were typed; a typing error rate of 0% and a confidence level of 80% were used. When more than one individual had the same LOD score, paternity was assigned to the spatially nearest individual. If an offspring lacked any potential pollen donor genotypes among the 257 candidate trees, we assumed that the pollen donor originated from outside the study plot.

### Data analysis

Genetic diversity indexes were calculated using the POPGENE32 software. ANOVA were performed using the generalised linear model (GLM) with maternal trees as random effects and stages (position) as fixed effects. Statistical analysis was performed using Excel (Microsoft, Redmond, WA, USA) and SPSS 16.0 (SPSS Inc., Chicago, IL, USA).

## Competing interests

The authors declare that they have no competing interests.

## Authors’ contributions

CQY participated in the study design, carried out the molecular and data analyses, and drafted the paper. YL participated in the study design, coordinated and supervised the analysis, and revised the manuscript. YHS, YFL, KQZ and RYH performed the field sampling. YHS and YFL participated in the data analyses and KQZ carried out the molecular studies. All authors read and approved the final manuscript.

## Supplementary Material

Additional file 1 Microsatellite markers used for the paternity analysis. Click here for file

Additional file 2 Outcrossing rates at three life stages. Click here for file

Additional file 3 Outcrossing rate at different positions within fruit. Click here for file
