# Supplementary material for: Selection occurs within linear fruit and during the early stages of reproduction in Robinia pseudoacacia
Source: BMC Evol Biol. 2014 Mar 21;14:53. doi: 10.1186/1471-2148-14-53 (PMC3998051; doi:10.1186/1471-2148-14-53)
Supplement: Additional file 1 — Microsatellite markers used for the paternity analysis. [file 1471-2148-14-53-S1.doc]

Additional file 1. Microsatellite markers used for the paternity analysis

|  |  |  |  |  | **Exclusion probability** | |
| --- | --- | --- | --- | --- | --- | --- |
| **Locus** | **Number of alleles** | ***H*_O_** | ***H*_E_** | ***PIC*** | **First parent** | **Second parent** |
| Rops06 | 16 | 0.916 | 0.823 | 0.801 | 0.485 | 0.657 |
| RP206 | 16 | 0.448 | 0.655 | 0.608 | 0.252 | 0.422 |
| Rops08 | 11 | 0.502 | 0.663 | 0.626 | 0.264 | 0.443 |
| RP109 | 14 | 0.586 | 0.773 | 0.745 | 0.400 | 0.581 |
| Rops05 | 15 | 0.718 | 0.834 | 0.814 | 0.508 | 0.677 |
| RP200 | 22 | 0.704 | 0.854 | 0.838 | 0.552 | 0.713 |
| Average | 15.67 | 0.6457 | 0.7669 | 0.7388 |  |  |
| Total |  |  |  |  | 0.9626 | 0.9957 |

Note: *H*_O_: observed heterozygosities; *H*_E_: expected heterozygosities; *PIC*: polymorphism information content.
