# Supplementary material for: Selection occurs within linear fruit and during the early stages of reproduction in Robinia pseudoacacia
Source: BMC Evol Biol. 2014 Mar 21;14:53. doi: 10.1186/1471-2148-14-53 (PMC3998051; doi:10.1186/1471-2148-14-53)
Supplement: Additional file 2 — Outcrossing rates at three life stages. [file 1471-2148-14-53-S2.doc]

Outcrossing rates at three life stages.

Outcrossing rate (%)

0

10

20

30

40

50

60

70

80

90

100

Aborted seeds

Mature seeds

Seedlings

Life stage

Outcrossing rate
