# Supplementary material for: Selection occurs within linear fruit and during the early stages of reproduction in Robinia pseudoacacia
Source: BMC Evol Biol. 2014 Mar 21;14:53. doi: 10.1186/1471-2148-14-53 (PMC3998051; doi:10.1186/1471-2148-14-53)
Supplement: Additional file 3 — Outcrossing rate at different positions within fruit. [file 1471-2148-14-53-S3.doc]

Outcrossing rate at different positions within fruit.

Outcrossing rate (%)

0

20

40

60

80

100

A

B

C

D

Position

Outcrossing rate
